# Supplementary figures and images for: A polycistronic transgene design for combinatorial genetic perturbations from a single transcript in Drosophila
Source: PLoS Genet. 2023 Jun 2;19(6):e1010792. doi: 10.1371/journal.pgen.1010792 (PMC10266610; doi:10.1371/journal.pgen.1010792)

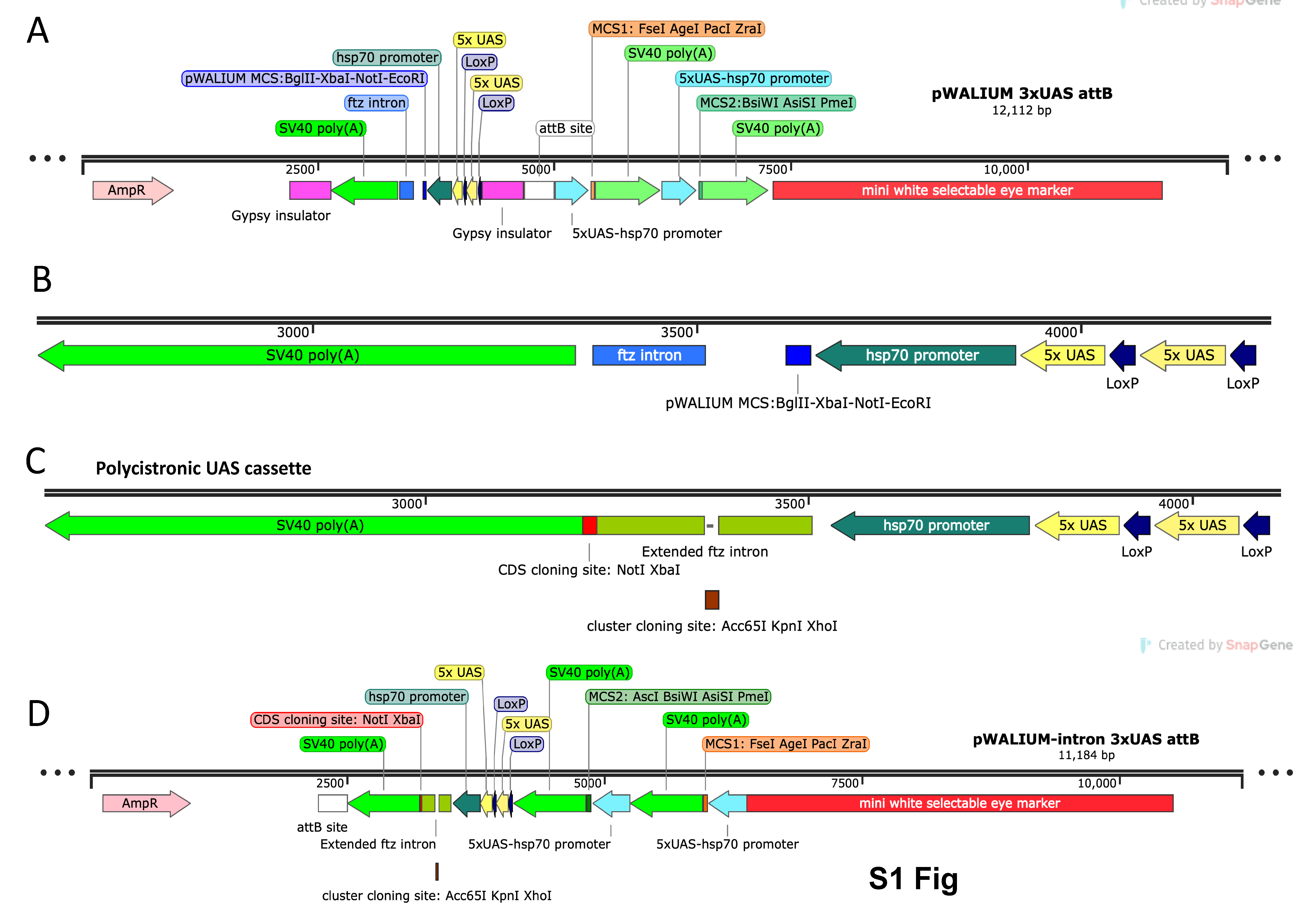

Supplement: S1 Fig — A. Map of our previously published pWALIUM 3xUAS attB vector, which includes three UAS cassettes, each with its unique multiple cloning site (MCS). B. Detailed map of the pWALIUM-derived cassette of pWALIUM 3xUAS attB, which was used to clone the test clusters in this study. C. The new polycistronic UAS cassette designed for intron-mediated expression of a short-hairpin cluster and a protein-coding sequence as a single transcript generated in this study. D. Map of the new pWALIUM-intron 3xUAS attB multigenic vector which includes the new cassette (C) along with two other standard cassettes (UAS-MCS1 and UAS-MCS2). Complete sequences of both plasmids are provided in S1 Data and S2 Data. (TIFF) [file pgen.1010792.s001.tiff]

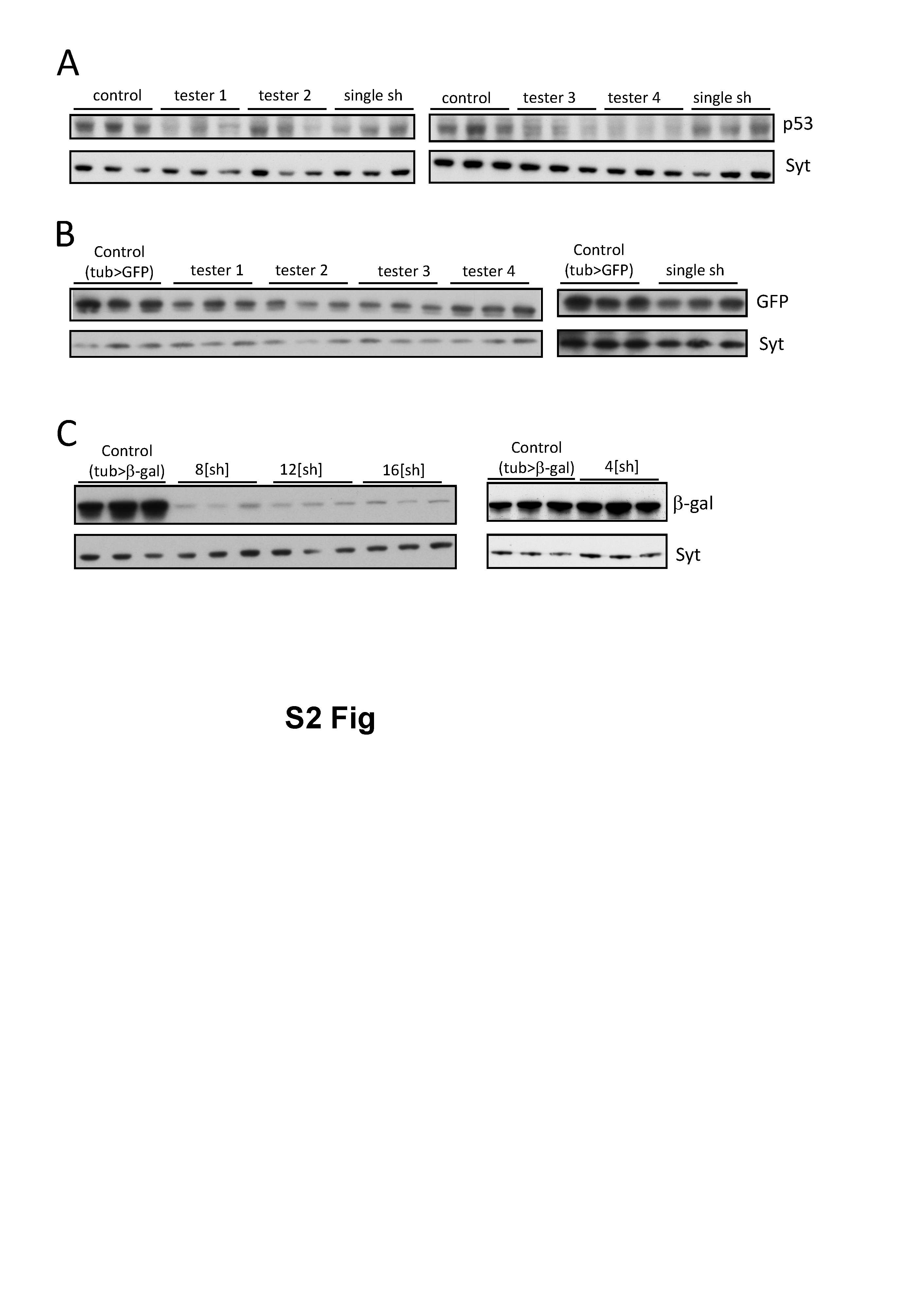

Supplement: S2 Fig — Western blot images used for generating the data presented in Fig 1C (A), 1D (B), and 2D (C). Syntaxin (Syt) was used as loading control. (TIFF) [file pgen.1010792.s002.tiff]

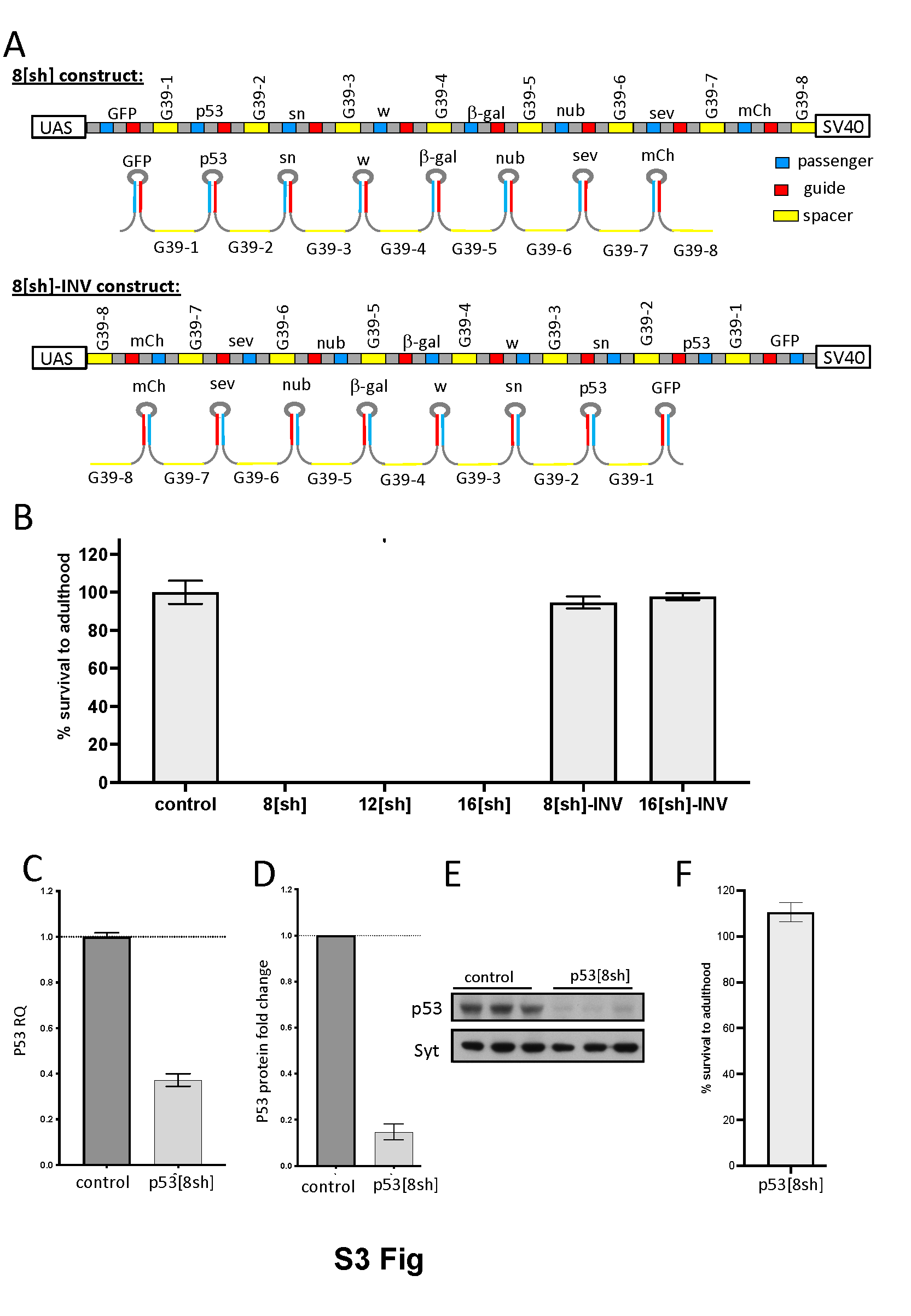

Supplement: S3 Fig — A. The inverted cluster design is illustrated using the 8[sh]-INV construct as an example. The inverted constructs are designed to generate the same number and sequence of hairpins when expressed but do not target any genes. B. Ubiquitous expression of the inverted 8[sh] and 16[sh] clusters does not result in organismal lethality. C-F. An 8-hairpin cluster targeting p53 with strong efficacy at the RNA (C), and protein level (D,E) does not result in organismal lethality upon ubiquitous expression during development (F). (TIFF) [file pgen.1010792.s003.tiff]
