# Supplementary material for: A polycistronic transgene design for combinatorial genetic perturbations from a single transcript in Drosophila
Source: PLoS Genet. 2023 Jun 2;19(6):e1010792. doi: 10.1371/journal.pgen.1010792 (PMC10266610; doi:10.1371/journal.pgen.1010792)
Supplement: Attachment — Submitted filename: Response to Reviewers.pdf [file pgen.1010792.s009.pdf]

Reviewer #1:

Overall assessment: In this manuscript the authors present a new tool for combinatorial knock down and overexpression of genes in Drosophila. Although the ability to model complex human diseases in the fly with this tool is impressive, the work represents only a modest advance over the author's own previously published tools. "We have previously demonstrated that eight genes can be effectively knocked down from a single synthetic short-hairpin (sh) cluster". How much of an advance is the new system from 8 genes? This paper is a further elaboration/confirmation of an already existing technology developed by this group. As they point out in the introduction, they had already created a "multigenic vector that contained three UAS cassettes for Gal4-mediated transgene expression..." PMID: 3113132. Furthermore, it is not at all clear which elements of the vector design are new to this report. It would be very helpful to have vector maps of the old and new versions, where the authors highlight what is new.

As far as I can tell, the advances described in this paper are: 1) Double the number of shRNAs possible to knockdown using a single transgene. 2) T2A-mediated expression of ORFs 3) Placing the shRNAs in the ftz intron. The authors also performed some nice experiments to show that position of the shRNA in the array does not affect knockdown efficiency.

The revised manuscript communicates the novelty and technical advancement of the work much more clearly. Briefly, the key advance of our new tool is that it consolidates genetic manipulations that require all three UAS cassettes of our original multigenic vector into a single polycistronic UAS transgene. This has two critical advantages over the previous system:

1) Consolidating multiple UAS transgenes into a single polycistronic one frees up two of the UAS cassettes of the multigenic vector, further increasing the number of transgenic manipulations that can be performed from a single multigenic vector. For instance, the remaining two UAS cassettes can be used to clone other transgenes like GFP, FLP recombinase, reporter constructs, or additional polycistronic transgenes.

2) The polycistronic transgene design can also be used to perform combinatorial genetic manipulations without the need for a multigenic vector. It can be adapted for use with the standard, single-cassette UAS vectors typically used for transgenesis, including those using QUAS system, FLP-Out method, and other commonly used promoters like actin, tubulin, heat-shock, etc.

To clarify which design elements are new to this report, we have included maps of the original multigenic vector and the new version designed for polycistronic transgene expression in Supplementary Figure 1. Complete plasmid sequences and annotated maps are also provided as supplemental files.

<u>Other comments:</u>

"Notably, p53 shRNA expression from all four 4[sh] clusters resulted in strong p53 knockdown, while the same sequence as a single hairpin did not (Figure 1C), indicating that short hairpins can be more effective in the context of a synthetic cluster. Whether this phenomenon generally applies to other short hairpins remains to be determined.

This is a very interesting finding if can be generally applied. It would strengthen the paper to show by qPCR or some quantitation of the phenotypes that for example, the other hairpins included in the UAS-tester strains produce less knockdown when expressed singly.

The other hairpins in the UAS-tester strains provided a comparable level of knockdown when expressed singly. An example is shown in Figure 1D, where there is no statistically significant difference in the level of knockdown across the four tester clusters and the single GFP hairpin. We had similar observations in our comparisons of other single hairpins versus longer clusters in cases where single hairpins were available as transgenic flies. We clarified this point in the revised manuscript. Although our observations with the p53 hairpin were not generalizable, it is important for future users of this design to be aware of the possibility that some hairpins could be more effective when expressed as part of a cluster. We also emphasize that, from a technology development perspective, the critical point Figure 1 demonstrates is that hairpin expression from a synthetic hairpin cluster <u>does not</u> reduce its efficacy.

“Despite these efforts, ubiquitous expression of the longer, 8[sh], 12[sh] and 16[sh] clusters during development resulted in organismal lethality” This is worrisome, as it is not clear whether the effect is due to synthetic lethality from target genes or possibly some interference with the endogenous siRNA/miRNA machinery. If the latter, this could confound any analysis of a disease phenotype. Although the authors show that reversing the sequences of the hairpin alleviates the toxicity, this does not rule out disruption of steps involving binding to an mRNA.

To address this point, we used an 8-hairpin cluster targeting the *p53* gene with 8 different hairpin sequences as another control. As *p53* is not required during development to produce viable offspring, any potential lethality associated with the expression of this cluster would be due to cluster toxicity rather than gene knockdown. This cluster provided strong p53 knock-down at the protein and RNA level, but its ubiquitous expression during development did not result in lethality. These results, now presented in Supplementary Figure 3C-F, further support our findings with the inverted clusters that long cluster expression is not inherently toxic to the organism.

Figure 1 (or supplemental) should include the western blots that were the bases for panels C and D

These are now provided in Supplementary Figure 2.

Figure 4 suggests that the ftz intron is not necessary for the processing of the shRNA clusters and does not improve knockdown. Maybe worth testing in a larger cluster to see if it has more of an effect there.

The ftz intron was included in the design to facilitate co-expression of a hairpin cluster and protein-coding sequences as a single transcript. Figure 4B shows that the presence of the intron does not negatively affect the level of knock-down, demonstrating that it can be safely used as a structural element in the polycistronic design. We clarified this point in the revised manuscript.

Reviewer #2:

In this paper, Teague and coworkers present a method allowing for multiplexed cell-type specific, as well as temporally controlled genetic manipulations in Drosophila. The method is based on previously published work, **however with substantial and very useful updates.** Central to the method is the integration of an extended array of short hairpins (used for gene knockdown), with a T2A-mediated, polycistronic protein expression strategy into a single transgene. Such a multigenic vector allows the concomitant repression and activation of gene expression as well as cell labeling, and hence, generation of complex disease models or the recapitulation of multilayered developmental processes in Drosophila.

The manuscript is well written, and the data presented is solid, interesting, and conclusive. Overall, the **described method is of sufficient novelty and technical advancement making the paper suitable for publication in PLOS Genetics**. A few minor comments are listed below that may enhance the paper:

The Figure panels Figure 3 are labelled as panel A, B, C, D, D (used twice), and E. They should be corrected to A-F as used in the text.

Corrected.

In Figure 4 (Panels A, C, and E) the architecture of the used vectors is depicted. To allow the reader to adapt the design for their own research, the details given by the authors are not sufficient, also in combination with the detailed Materials and Methods section. There, the authors cite two papers, (References 11 and 16) where the used vector design is described with higher granularity. And even in one of these papers the authors cite yet another paper (Ni et al, 2011) regarding the original vector that was obviously used. It is not very helpful to constantly talk of a “multigenic vector” - such things need a precise name regarding the used vector, an accession number or similar. For this publication, the authors should at least show drawings with a similar if not higher amount of detail (as in the cited papers) to support the adaptability and use of their method in different research projects. The reader should not be forced to read other papers to understand the underlying design nor to make the necessary adaptations to get a view on the multigenic vector(s) used for the current manuscript.

We now include maps of the original multigenic vector (**pWALIUM 3xUAS attB**) and the new version designed to allow polycistronic transgene expression **(pWALIUM-intron 3xUAS attB)** in Supplementary Figure 1. We consistently used these names to refer to the vectors throughout the manuscript and clearly stated which plasmid and multiple cloning sites were used for each construct. We also provide complete sequences of the two vectors and annotated maps as supplemental data files.

In the introduction, the authors mention, that they expanded the number of shRNAs expressed from a single cluster to 16. However, the expression of longer clusters (8, 12, 16) leads to lethality. Although the authors find ways (using tub-gal80ts) to use longer arrays and evaluate their knockdown efficiency, the sentence should be removed from the introduction as such long hairpins can only be used in specific contexts. This limitation of the method should also be debated in the discussion section.

We agree that this is a critical point we could have emphasized better in the original manuscript. We have addressed it by clarifying the potential uses for the longer clusters throughout the manuscript. We acknowledge that potential synthetic lethality due to the simultaneous knock-down of 8-16 genes could limit the utility of longer arrays. We discuss that while feasible, simultaneous knock-down of that many genes would be a niche use for this technology, especially ubiquitous knockdown at the whole organismal level. On the other hand, targeting a few genes with multiple different hairpins each to ensure knockdown would be a more broadly relevant potential application, a transgenic, *in vivo* equivalent of siRNA pools used for transfection experiments in cell-based assays. (e.g., the 16-hairpin cluster targeting 4 genes in Figure 3). We also discuss the use of long clusters as an in vivo hairpin discovery tool to evaluate the efficacy of hairpins targeting multiple genes at once to identify optimal sequences for subsequent studies. These additional applications significantly broaden the utility of this technology.

Reviewer #3:

In this manuscript, Teague et al., report a new platform to co-knockdown and co-overexpress multiple genes simultaneously from a single transgenic construct in Drosophila melanogaster. In a previous paper, the authors generated a multigenic vector that contained three UAS cassettes which together can mediate overexpression or knockdown of multiple genes (e.g. overexpression of 2 genes while knocking down 8). In this study, the authors further expand this technology by incorporating an intron-mediated shRNA expression and a T2A-based multi-protein expression systems into their platform. This versatile platform will allow users to manipulate multiple genes using a single transgenic plasmid. This will permit fly researchers to test various hypotheses that relate to interaction of multiple genes. The proof of principle experiments have been carried out with rigor, and their method has been explained well in the text. While the organism lethality they observed when they knocked down too many genes simultaneously may limit the application of this technology for certain applications, I believe **the field would benefit from the publication of this paper in PLoS Genetics**. I only have some minor comments that the authors can consider when finalizing the manuscript.

Major Comments:

None

Minor Comments:

1) I recommend the authors to italicize the word "Drosophila" and "in vivo" throughout the text (done in some places but not in a consistent manner).

Corrected.

2) While some papers refer to T2A as an "self-cleaving peptide", I believe "ribosomal skipping peptide" will be a more accurate term (e.g. in abstract).

Corrected.

3) In the introduction, the authors say "Hundreds of transgenic Drosophila Gal4 lines with…" but there are "Thousands" of these types of lines available from public stock centers.

Corrected.

4) There is one place that refers to "Gal80ts" as "gal80ts" in the introduction, which needs to be fixed.

Corrected.

5) Reference [8] is talking about a collection of UAS lines, rather than GAL4 lines. Hence, this paper is inappropriate to cite here. I recommend the authors cite papers like PMID 23063361, 23063363, 23063364, 29565247, 33092520 and/or https://www.biorxiv.org/content/10.1101/198648v1.

Corrected.

6) In the last sentence of the introduction, I recommend the authors to change "standard P-element-based vectors" to "standard P-element and phiC31-based vectors".

Corrected.

7) Gene and allele names need to be italicized in many places (e.g. p53, TP53, APC, SMAD4, SMAD2, dRAS[G12V], Chico), unless the authors are referring to the encoded protein.

Corrected.

8) “TRIP” should be written as “TRiP” in some places.

Corrected.

9) The authors conclude that the organism level lethality observed when expressing multiple genes is likely an 'additive response to the simultaneous knockdown of multiple genes'. Considering that each of the genes the authors are knocking down here seems to be non-essential genes, do the authors think there is some kind of a ‘synthetic lethality’ that takes place when these genes are co-knockdown? Also, the authors may want to clearly document that while this type of organism level may limit the utility of this technology for certain applications in the discussion section of this paper.

While we included as many exogenous and non-essential genes as possible, some of the hairpins in these clusters do target essential genes. We sought to minimize lethality by choosing hairpins that did not produce a lethal phenotype upon ubiquitous expression as single transgenes. Still, we could not eliminate the possibility that the ubiquitous knock-down of multiple such genes, even at moderate levels, may lead to a synthetic lethal phenotype. We now more clearly discuss the limitations of knocking down a large number of genes, especially ubiquitously in a whole animal.

We acknowledge that potential synthetic lethality due to the simultaneous knock-down of 8-16 genes could limit the utility of longer arrays. We also stress that while feasible, simultaneous knock-down such a large number of genes would be a niche use for this technology, especially ubiquitous knockdown at the whole organismal level. On the other hand, targeting a few genes with multiple different hairpins each to ensure knockdown would be a more broadly relevant potential application, a transgenic, in vivo equivalent of siRNA pools used for transfection experiments in cell-based assays. (e.g., the 16-hairpin cluster in Figure 3, which targets 4 genes with 4 different hairpins each). We also discuss the use of long clusters as an in vivo hairpin discovery tool to evaluate the efficacy of hairpins targeting multiple genes at once to identify optimal sequences for subsequent studies. These additional applications significantly broaden the utility of this technology.

10) In the final paragraph of the discussion section, the authors refer to ‘enhanced off-target effects’ as one of the drawbacks of CRISPR based technology when performing multiplexed gene editing. However, multiplex shRNA approach the authors take in this study also has the same risk (i.e. as the number of shRNA increases, the risk of having an off-target effect also increases). Hence, I feel the authors also should discuss this as a potential limitation of this study, and additional control experiments (e.g. rescue or validation experiments) need to be performed to make strong conclusions regarding the additive or synergistic interactions between multiple genes when using this type of approach.

We expanded this section to acknowledge this excellent point as a shared limitation of both technologies and the importance of using additional control experiments to carefully validate these tools before they are used for biological experiments.
